# Potential Role of Epstein–Barr Virus in Oral Potentially Malignant Disorders and Oral Squamous Cell Carcinoma: A Scoping Review

**DOI:** 10.3390/v14040801

**Published:** 2022-04-13

**Authors:** Rifat Rahman, Divya Gopinath, Waranun Buajeeb, Sopee Poomsawat, Newell W. Johnson

**Affiliations:** 1 Menzies Health Institute Queensland, School of Medicine and Dentistry, Griffith University, Gold Coast, QLD 4222, Australia; rifat.rahman@griffithuni.edu.au (R.R.); n.johnson@griffith.edu.au (N.W.J.); 2 Clinical Oral Health Sciences Division, School of Dentistry, International Medical University, Kuala Lumpur 57000, Malaysia; 3 Department of Oral Medicine and Periodontology, Faculty of Dentistry, Mahidol University, Bangkok 10400, Thailand; waranun.bua@mahidol.edu; 4 Department of Oral and Maxillofacial Pathology, Faculty of Dentistry, Mahidol University, Bangkok 10400, Thailand; spoomsawat@gmail.com; 5 Faculty of Dentistry, Oral and Craniofacial Sciences, King’s College London, London WC2R 2LS, UK

**Keywords:** Epstein–Barr virus (EBV), oral squamous cell carcinoma (OSCC), oral potentially malignant disorders (OPMD), latent membrane protein 1 (LMP-1)

## Abstract

Though the oral cavity is anatomically proximate to the nasal cavity and acts as a key reservoir of EBV habitation and transmission, it is still unclear whether EBV plays a significant role in oral carcinogenesis. Many studies have detected EBV DNA in tissues and exfoliated cells from OSCC patients. However, very few studies have investigated the expression of functional EBV proteins implicated in its oncogenicity. The most studied are latent membrane protein 1 (LMP-1), a protein associated with the activation of signalling pathways; EBV determined nuclear antigen (EBNA)-1, a protein involved in the regulation of gene expression; and EBV-encoded small non-polyadenylated RNA (EBER)-2. LMP-1 is considered the major oncoprotein, and overexpression of LMP-1 observed in OSCC indicates that this molecule might play a significant role in oral carcinogenesis. Although numerous studies have detected EBV DNA and proteins from OSCC and oral potentially malignant disorders, heterogeneity in methodologies has led to discrepant results, hindering interpretation. Elucidating the exact functions of EBV and its proteins when expressed is vital in establishing the role of viruses in oral oncogenesis. This review summarises the current evidence on the potential role of EBV in oral oncogenesis and discusses the implications as well as recommendations for future research.

## 1. Introduction

Seroprevalence data reveal that EBV (Human herpesvirus 4) has infected up to 90% of the global population and EBV seroprevalence rates among children and adolescents seem to be declining in some populations, indicating that the population at risk of complex primary EBV infection may be increasing [1,2]. After primary infection, EBV establishes latency in a minor proportion of B lymphocytes located in the lymphoid tissues, including those of the head and neck, notably Waldeyer’s ring, as well as in the lining epithelia of the naso and oropharynx, and in salivary glands where it replicates. It is often shed in secretions of the upper aerodigestive tract, without obvious signs or symptoms in most people [3].

EBV is strongly implicated in the causation of nasopharyngeal carcinoma (NPC), gastric carcinoma and lymphoproliferative disorders such as Burkitt’s and Hodgkin’s lymphoma. It is regarded as driving the epithelial hyperproliferation and abnormal keratinization of oral hairy leukoplakia, a benign lesion mainly found in immunocompromised patients, and B cell lymphomas [4].

EBV is part of the normal oral flora and recent investigations confirm that it is clinically associated with infections of gingivae, periodontal ligament, tooth pulp and periapical tissues [1]. Many studies have reported the presence of EBV in normal oral epithelium, oral potentially malignant disorders (OPMD), including oral lichen planus (OLP) [5], and oral leukoplakia (OL) [6] as well as in oral squamous cell carcinoma (OSCC). [5] EBV nucleic acid has been detected routinely in OPMD with a prevalence ranging from 6.2% to 72.2%. [5,6,7,8,9,10,11]. EBV proteins have been detected in SCC from the maxillary sinus [7]. Here, we summarise the current evidence on the association of EBV with OPMD and OSCC and throw insights into its potential role in oral carcinogenesis.

## 2. Characteristics of HHV4, the Epstein–Barr Virus

**Figure 1:**
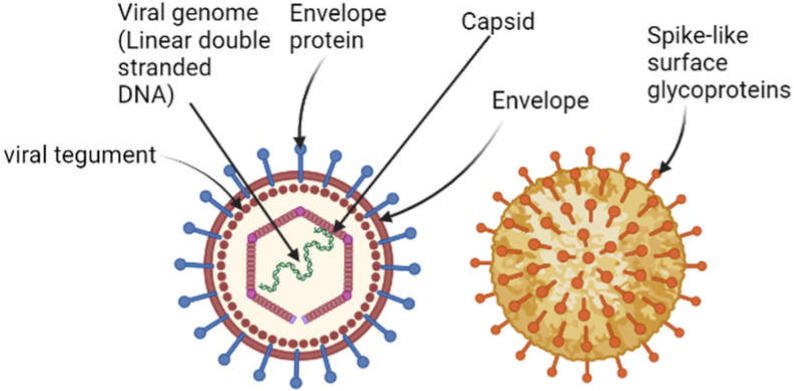
Structure of EBV virion (Image created by biorender.com, accessed on 23 January 2022).

The EBV is a gammaherpesvirus, with a linear, double-stranded DNA genome of 170–185 kb encoding for more than 85 genes [8]. The EBV genome harbours a series of 0.5-kb terminal direct repeats located at both ends and internal repeat sequences that separate the whole genome into long and short distinctive domains which encode the proteins [9]. The nucleocapsid of EBV is made up of 162 capsomeres covered by a viral envelope derived from host membranes. The space between the nucleocapsid and the envelope is called the tegument. The outer envelope harbours the surface glycoproteins that form “spike-like” protrusions [10] (Figure 1).

EBV enters epithelial cells and lymphocytes by an interaction between these viral glycoproteins and cellular receptors: viral entry occurs by direct fusion of the envelope with host cell membranes. Following entry, the linear viral genome changes to circular because of the merging of the terminal direct repeats at both the ends of the linear DNA [11]. The EBV genome inside the replicated cell is maintained as an extrachromosomal episome.

## 3. EBV Proteins and Their Functions

**Figure 2:**
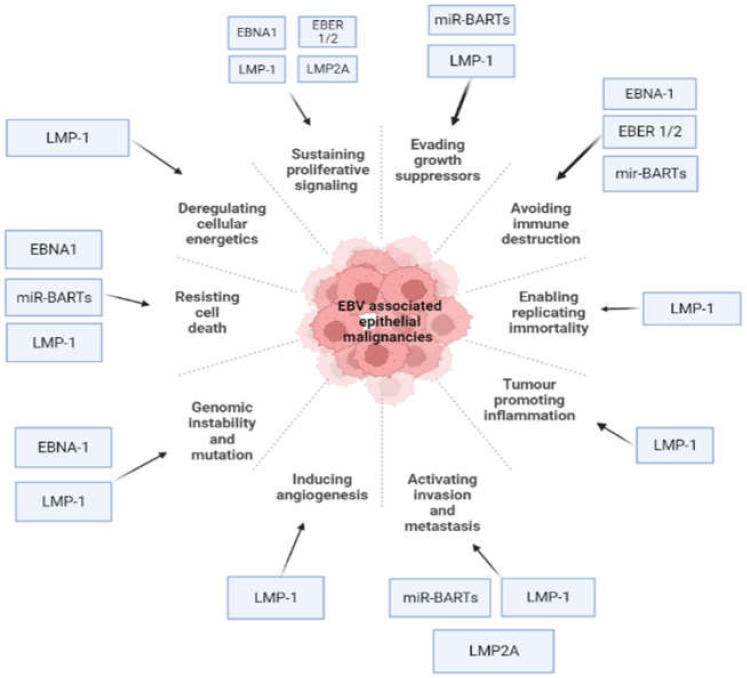
Epstein–Barr virus (EBV) latent genes target cancer hallmarks of epithelial malignancies (Image created by biorender.com, accessed on 23 January 2022).

EBV encodes a series of proteins expressed at different periods following the infection of B cells. These include Epstein–Barr nuclear antigens (EBNA-1, EBNA-2, EBNAs-3A,3B, 3C, and EBNA-LP), the viral BCL-2 homolog, BamHI-H rightward open reading frame 1 (BHRF1) and the latency membrane proteins-1 and -2 (LMP-1 and LMP-2). It also contains two non-coding RNAs (EBER1 and EBER2) and two sets of miRNAs encoded within the BamHI—the rightward transcripts (BARTs) and the BHRF1 locus (BHRF1 miRNAs) [12,13]. These EBV products have been investigated for their potential role in oncogenesis by facilitating the important hallmarks of malignancy [12,14] (Figure 2).

EBNA-1 is the main protein that maintains latency before the virus enters its replication stage [15,16,17]. The primary function of EBNA-1 in latent infection is to facilitate viral genome replication and to mediate genome segregation into daughter cell nuclei. EBNA1 is a homo-dimeric protein and binds site-specifically to a DNA sequence 16 bp in length [18,19,20]. It is now known that EBNA1 can link regions of DNA to which it binds, and often forms a loop between the family of repeats (FR) and the Dyad of Symmetry (DS). EBNA1’s ability to link DNA correlates with its support for replication and transcription [20,21]. EBNA1 also regulates a subset of signalling pathways in the host cells and thereby contributes to the survival and proliferation of EBV-infected cells. EBNA-1 binds to cellular promoters and upregulates STAT1 (signal transducers and activators of transcription 1), whose expression along with an increase in major histocompatibility complex class I and II downregulates various pathways, including tumour growth factor-β (TGF-β) signalling pathways and the canonical NF-κB pathway [17,19,20].

EBNA-2 is a transcriptional-coactivator that influences the expression of viral genes in the latency period and regulates many cellular genes by binding to super-enhancer regions in the cell chromatin, ultimately leading to cell cycle entry for proliferation. EBNA-2 mediates some of this regulation through interactions with human transcription factors, including SPI1 (PU.1), RBPJ, EBF1 and many more [22,23]. EBNA2 activates transcription through multiple interactions between its acidic transactivation domain (TAD) and ATP-dependent remodellers histone acetyltransferases and elements of the pre-initiation complex [24].

EBNA-LP, also known as EBNA-5, is the first viral latency-associated protein produced after the EBV virus infects a cell. It acts with EBNA-2 to lead the cell into the cell cycle [25,26]. EBNA-LP has been shown to enhance EBNA2 mediated transactivation of LMP-1 [27]. It has been shown to have multiple interactions with host genes involved in tumour suppression, apoptosis and cell cycle regulation; however, there is no consensus in the literature regarding the genes affected [28].

The EBNA-3 family, consisting of EBNA-3A, EBNA-3B, and EBNA-3C, function as transcriptional regulatory proteins through their interactions with DNA binding proteins and other auxiliary transcription factors, as an alternative to directly binding to DNA [29]. Though a large number of such interacting proteins have been identified, RBPJ (or CBF1), a downstream regulator of the Notch signalling pathway, is the most established transcription factor crucial for EBV-induced host transformation [29,30]. All EBNA-3 proteins have similar gene structures, are comparably regulated and share a common binding site of RBPJ [31].

Among the latency-associated proteins, LMP-1 is the dominant oncoprotein, commonly expressed in EBV-related cancers [12,32]. LMP-1 interacts with molecules that mediate signals from tumour necrosis factor (TNF) receptors, and thus activates multiple signaling pathways, including NF-κB, JNK–p-38, ERK–MAPK, PI3K–AKT and JAK-STAT [33]. Many reports have suggested that the activation of these signal transduction pathways facilitates a plethora of downstream effects, including the expression of adhesion molecules and growth factor receptors, cell proliferation, anti-apoptosis and angiogenesis [34,35]. Furthermore, it has also been shown to upregulate telomerase activity via cMyc induction and promote the migration of tumour cells by inducing the activation and secretion of different matrix metalloproteinases [36]. The protein has also been shown to induce epithelial–mesenchymal transition in NPC as well as having cancer stem cell-like properties [37]. Previous studies have found the expression of LMP-1 in 40–90% of NPC [38,39,40,41,42]. LMP-1 has three functional domains, C-terminal activation regions (CTAR 1, CTAR 2, CTAR 3) [43]. CTAR 1 triggers NF-κB immortalization of infected cells via the activation of telomerase and blockage of apoptosis. It has also been shown to stimulate cell proliferation by activation of cyclin D1, cyclin E and EGFR signalling pathways. CTAR2 has been shown to trigger an AP-1 signalling cascade, which upregulates the expression of matrix metallopeptidase 9 (MMP-9). CTAR3 also triggers the JAK3/STAT signalling pathway, which enhances the transcription and expression of vascular endothelial growth factor (VEGF) [44].

## 4. Clinical Studies on the Association of EBV with OPMD and OSCC

Table 1 lists the findings regarding the detection of EBV DNA and its associated proteins from studies conducted in different parts of the world in normal mucosa, OPMD and OSCC using PCR, in situ hybridization, EBV genomic microarray (EBV-chip) and immunohistochemical techniques. EBV was not only found in OPMD and OSCC but also detected in the normal oral epithelium and can be regarded as part of the normal oral flora. In normal oral samples, the prevalence of EBV ranges widely, differing by the geographical location, gene target used, method of sample collection and detection techniques.

### 4.1. Prevalence of EBV Based on Geographical Location

Variation in prevalence of carriage of EBV DNA in formalin-fixed paraffin-embedded (FFPE) OSCC according to the geographical location was demonstrated in a Japanese study carried out in Okinawa and Sapporo. The study used PCR and detected EBV DNA in 76.6% of Okinawan and 38.1% of Sapporon cases [60]. Differences in EBV prevalence were also shown in three Thai studies, but these apparently used different methods of sample collection, selection of gene targets and detection techniques [46,51,56]. The study conducted by Acharya et al., in 2015, detected EBV DNA in exfoliated cells of 45% of OSCC cases using PCR in a Northeast Thai population. This was supported by a recent Thai study from the same population that revealed strong EBER-positive signals in FFPE OSCC tissues by in situ hybridization [46]. However, when Iamaroon et al., in 2004, used an in situ hybridization technique in the Northern Thai population, they found none of the 24 cases of OSCC to express EBER transcripts [56]. Thus, variations in EBV prevalence based on geographical location as well as detection techniques are observed in these studies [46,56].

### 4.2. Prevalence of EBV Based on the Method of Sample Collection

Among the techniques of sample collection, throat washings, oral smears and scraping methods seem to demonstrate a higher EBV prevalence with 20–90% in normal mucosa of healthy adults [59,67,68] being positive compared with biopsy specimens in normal mucosa (0–40%) [5,49,52,54,63,64,65,66,67]. In Japan, EBV DNA was identified in 90% of throat washings from healthy adults and in 38% of saliva samples from healthy children [59]. Since EBV is present as part of the normal oral flora in healthy people, immunosuppression allows EBV to easily infect oral epithelial cells as exemplified by oral hairy leukoplakia [69].

### 4.3. Prevalence of EBV Based on the Method of Detection

Because EBV is regarded as a useful tumour marker in certain neoplasms, laboratory testing of EBV and the identification of viral gene products have become critical. There are several diagnostic methods for EBV detection, including serological and molecular diagnostic methods, each with its own advantages and limitations. Although in situ hybridization (ISH) is the gold standard, detecting EBV with a 100% sensitivity, the molecular determination of viral DNA, RNA and EBV viral load is now being used in the clinical assessment of tumour-associated EBV infections [70,71,72]. Among the studies we reviewed, the presence of EBV nucleic acids in affected tissues was performed by in situ hybridization (ISH) and by PCR, and the detection of EBV-related proteins, including EBV nuclear antigen 2 (EBNA2) and the latent membrane proteins, were detected by immunohistochemical assays. As previously stated, the percentage of EBV-positive cases in NM, OPMD and OSCC varied among studies, and one of the most significant causes for this wide range may be the sensitivity of the method employed. Of the three EBV detection methods reported, we found that PCR yielded a higher EBV-positive detection rate than did ISH or IHC (Table 1). The reason for this may be that the target DNA is able to be amplified thousands of times by PCR and thus has a higher sensitivity. On the other hand, PCR is unable to provide information on the cellular localization of the virus.

### 4.4. Prevalence of EBV in Normal Mucosa, OSCC and OPMD

The detection rate of EBV has been reported to range from 0 to 92% in normal mucosa (Table 1) [5,6,58,63,73]. Overall, however, EBV has been shown to be more prevalent in OSCC than in normal mucosa [74]. The accumulated evidence so far remains inconclusive regarding the presence of EBV itself in OSCC, with results again ranging from 0 to 100% [5,57,75]. Three studies conducted in East Asia found high EBV prevalence in OSCC [55,74,76]. A study in the Netherlands found EBV DNA in 100% of OSCC using PCR [64]. This was supported by a study conducted in Spain, which showed the positivity of EBV DNA, using PCR, to increase from well differentiated to poorly differentiated OSCC [62]. In Asia, numerous studies have come from different parts of Japan with variable findings (0–85.7%) [45,46,48,60,61]. Two Japanese studies detected a high prevalence of EBV and suggested a causative role in OSCC [74,76]. In contrast, studies of subjects from North America, and North and West Europe reported lower EBV prevalence and concluded that the pathogenic role of EBV in OSCC is doubtful [67,75,77]. EBV seems to be more prevalent in OSCC than in OPMD [52,54,64] with a variation in results when different methods of detection are used.

Kikuchi et al., in 2016, detected LMP-1, the main oncoprotein of EBV, in OSCC, in oral epithelial dysplasia and in morphologically normal mucosa from tongue and gingival fibrous overgrowths. They found a significantly higher expression of LMP-1 in severe epithelial dysplasia than in OSCC [50]. They suggested that an increased expression of the latent infection gene in severe epithelial dysplasia could indicate a crucial role for the virus in the dysplasia-to-carcinoma sequence within the oral cavity. Conversely, a study conducted in Egypt reported that malignant oral mucosa expressed LMP-1 more evidently than oral epithelial dysplasia [53]. These findings are supported by our recent study in Thailand, as well as several studies conducted in Spain, Bosnia and Japan using immunohistochemistry [47,49,57,61]. On the contrary, another study conducted in Hungary did not find any LMP-1 expression in OSCC using the same method [54]. Since LMP-1 plays a key role in the malignancies known to be driven by EBV, and since previous studies have such conflicting results, further work on larger samples with the best techniques is required to clarify the role of LMP-1 in the dysplasia-carcinoma sequence of the oral cavity.

A gene exerts its effects by transcribing DNA into mRNA, which is then translated into a protein, the final effects of the gene’s action taking place in the cytoplasm [78]. Hence, increased LMP-1 expression in the cytoplasm of OL with dysplasia and OSCC is indicative of the LMP-1 gene in the functional state during the early and late events of oral carcinogenesis. It does not mean, however, that the protein is necessarily oncogenic: association does not prove cause and effect. Aetiological agents and pathogenic pathways can and do vary from case to case. In our study, we were the first to analyse the intracellular localization of LMP-1 in normal oral mucosa, OL with and without dysplasia and OSCC [49]. In normal oral mucosa, cytoplasmic staining alone was not observed. Nuclear staining was observed in all the cases, but only a few cases showed nuclear plus cytoplasmic staining [49]. In OSCC, cytoplasmic staining alone and nuclear plus cytoplasmic staining were considerably more intense compared to those of normal mucosa, and OL with and without dysplasia. Prominent golden-brown colour staining in the cytoplasm of OSCC was prominently observed compared to normal mucosa. Changes in the localization of LMP-1 protein observed in OSCC suggest a potential role in oral carcinogenesis. An increased expression of LMP-1 was detected in 81.8% (18 out of 22) of OSCC, higher than the 62.5% (10 out of 16) of epithelial dysplasia in the study by Shamaa et al [53].

A meta-analysis by She et al., published in 2017, attempted to pool studies on the association of detection of EBV in OSCC [79]. However, the results have to be interpreted with caution, as the confidence intervals were very wide in all the analyses presented. Moreover, high heterogeneity was detected in overall and subgroup analyses, which adds to the uncertainties. Regardless of the statistical evidence, our review is in agreement with the previous meta-analysis in that EBV is more prevalent in the OSCC samples than in normal controls.

## 5. Molecular Mechanisms of EBV Infection Involved in Oral Carcinogenesis

The mechanism by which EBV takes part in carcinogenesis may vary for each malignancy, but some common features are notable. EBV is found to remain in a latent state within its associated malignancies. EBV latency permits the persistent expression of viral oncogenes while simultaneously preventing immune detection and cytopathic effects during the replicative phase of the viral life cycle [80]. EBV reactivation can be triggered by two immediate-early (IE) trans-activators, namely Zta and Rta, after various stimulating factors, including sodium butyrate, 12-O-tetradecanoylphorbol-13-acetate (TPA), anti-Ig, and TGF-β. Running these two IE proteins at the same time turns on the whole lytic viral cascade of gene expression and EBV replication [81]. Even after the loss of EBV, it has been reported that delayed differentiation and enhanced invasiveness were retained in epithelial cells, demonstrating that a stable epigenetic reprogramming occurred after EBV infection [82,83].

Cyclin D1 is a key cell-cycle regulatory protein that promotes G_1_/S transition in cells [84]. Disorders in cell cycle control can lead to uncontrolled proliferation, a hallmark of malignancy and observed in OSCC [85]. Overexpression of cyclin D1 is not only observed in OSCC [86,87] but is also seen in OPMDs and oral epithelial dysplasia [88,89]. This is supported by a recent immunohistochemical study which showed overexpression of cyclin D1 protein from epithelial dysplasia to OSCC: a proportional increase in the percentage of cyclin D1 expression with an increase in the histopathological grade, i.e., from well differentiated to poorly differentiated OSCC [90]. These findings indicate that cyclin D1 deregulation is a major, and early, event in the neoplastic process. Cyclin D1 overexpression is closely associated with EBV infection. By upregulating the expression of cyclin D1 via the NF- κB signalling pathway and downregulating the expression of p16, LMP-1 promotes uncontrolled cell proliferation by accelerating the transition of the cell cycle from G1 to S phase [91].

**Figure 3:**
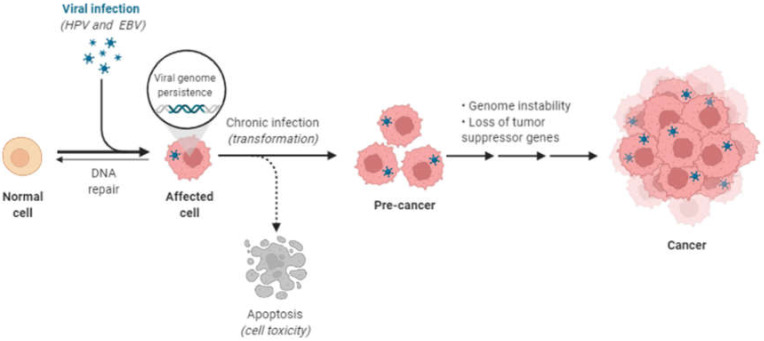
The process of carcinogenesis as a result of viral integration into the host genome (Image created by biorender.com, accessed on 23 January 2022).

Some authors have found coinfections of human papillomavirus (HPV) and EBV in OSCC [4,92]. High-risk HPV infection alone is not enough to initiate neoplastic transformation in normal human epithelial cells, including those of the head and neck region [93]. Coinfection by multiple oncogenic viruses may be a significant risk factor in the development of OSCC [92,94] (Figure 3). Experimentally, an EBV infection has been shown to promote the invasive phenotype and delay differentiation in epithelial cells expressing HPV16 E6 and E7 oncogenes [82,92]. Toll-like receptors (TLRs) play an important role in the early innate immune response against invading pathogens by sensing a microorganism [95]. LMP-1 is a potent inhibitor of TLR9 transcription, which can enhance the aforementioned superinfection [95]. Further studies on coinfected cells are required as the combined impact of both HPV and EBV may increase the potential for carcinogenesis compared to normal cells [92].

## 6. Conclusions

EBV is clearly oncogenic in NPC and some lymphomata. Although there is substantial literature describing the presence of EBV nucleic acid and/or EBV proteins in normal, dysplastic and neoplastic oral mucosa, the findings have not been conclusive regarding the oncogenicity of this ubiquitous virus for oral cancer. This is only partly because of the inconsistency of results and variations in the technology applied. Even if the presence of viral markers was more consistent, this would not constitute proof. It cannot be said too often that association does not prove cause and effect. We must always bear in mind the Bradford Hill principles and the strict criteria for causation [96]. Whilst further epidemiological studies using standardised techniques will be helpful, there is a need for mechanistic, hypothesis-driven experiments in tissue culture models and animals in order to move our understanding forward. Should solid evidence emerge, we might look forward to vaccination of the population, for which early research is encouraging, to contribute to the control of oral cancer.

## Figures and Tables

**Table 1:** Clinical studies on detection of EBV in OPMD and OSCC.

Author	Year	Study Location	Total No. of Samples	Detection Method	Sample Type	Marker	Major Findings	Reference
Vanshika S, et al.	2021	India	108 OSCC	PCR	FFPE	EBV DNA	EBV DNA detected in 27.8% (30 out of 108)	[45]
Heawchaiyaphum C, et al.	2020	Thailand	165 OSCC	EBV detection by PCR and EBER by ISH	FFPE	EBNA1, LMP1	EBV DNA detected in 41% (68 out of 165) (To further confirm the infection of EBV in OSCC, EBER-ISH was performed. Strong EBER-positive signals were detected in epithelial cells of OSCC tissues.	[46]
Al-Thawadi H, et al.	2020	Bosnia	64 OSCC	PCR, IHC	FFPE	LMP-1	LMP-1 detected in 78% (50 out of 64) by PCR. 76% (35 out of 46) by IHC	[47]
Naqvi SU, et al.	2020	Pakistan	58 OSCC	PCR	FFPE	EBV DNA	EBV DNA detected in 26% (15 out of 58)	[48]
Reddy SS, et al.	2017	India	75 OPMD (25), OSCC (25), NM (25)	IHC	FFPE	LMP-1	LMP-1- detected in 8% (2 out of 25) OSCC, 8% (2 out of 25), OPMD, 8% (2 out of 25) NM	[32]
Rahman R, et al.	2019	Thailand	115 NM (10)OL without dysplasia (27) OL with Dysplasia (42) OSCC (36)	IHC	FFPE	LMP-1	LMP-1 detected in NM (26.36%), OL without dysplasia (28.03%), OL with dysplasia (34.15%), OSCC (59.67%)	[49]
Kikuchi K, et al.	2016	Japan	248 150 OSCC, 83 dysplasia (mild: 22, moderate: 43, severe: 18), 15 NM	PCR	FFPE	EBV DNA (EBNA-2), LMP-1)	LMP-1 detected in Normal gingiva 33.3%, Mild dysplasia 45.5%, Moderate dysplasia 4.7%, Severe epithelial dysplasia 44.4%, OSCC 10.7% EBV DNA- (EBNA-2) detected in normal gingiva 71.3%, Mild 22.7%, moderate dysplasia 53.5%, Severe epithelial dysplasia 66.7%, OSCC 52%.	[50]
Acharya S, et al.	2015	Thailand	185 (91 OSCC, 94 NM)	PCR	Exfoliated cancer cells	EBV DNA	EBV DNA detected in oral exfoliated cells 45.05% of OSCC patients 18.08% of NM	[51]
Bagan L, et al.	2016	Spain	71 (12 OSCC, 12 OPMD, 47 NM)	PCR	Saliva	EBV DNA	EBV DNA detected in saliva NM (40.4%) OSCC group (58.3%) OPMD group (41.7%)	[52]
Shamaa AA, et al.	2008	Egypt	58 (22 OSCC, 16 epithelial dysplasia, 20 NM)	IHC	FFPE	LMP-1	LMP-1 detected in NM-negative, Epithelial dysplasia- 62.5% (10 out of 16), OSCC—81.8% (18 out of 22).	[53]
Kis A, et. al.	2009	Hungary	293 (65 OSCC, 44 OL, 116 OLP, 68 NM)	PCR & IHC	FFPE	EBV DNA	EBV DNA detected in NM-19.1%, OSCC-73.8%, OL-29.5%, OLP lesions-46.6% In OSCC, LMP-1 expression was not detected.	[54]
Yen CY, et al.	2009	Taiwan	57 OSCC	EBV genomic microarray (EBV-chip)	FFPE	EBV DNA	EBV DNA detected in 85.7%) of biopsy specimens of OSCC indicating high rate of EBV infection	[55]
Bagan JV, et al.	2008	Spain	20 (5 PVL, 10 OSCC, 5 NM)	PCR	FFPE	EBV DNA	EBV DNA detected in 60% Proliferative verrucous leukoplakia 40%- OSCC 0%- NM	[6]
Iamaroon A, et al.	2004	Thailand	185 (91 OSCC, NM 94)	ISH	FFPE	EBV RNA	Expression of EBV RNA studied in 24 cases of OSCC. None of OSCC expressed RNA *(EBER)* transcripts.	[56]
Sand LP, et al.	2002	Sweden	119 (29 OSCC, 23 OLP, 67 NM)	PCR	FFPE	EBV DNA	EBV DNA detected in Oral mucosa-7.3% OLP-26.1% OSCC-37.9%	[5]
Gonzalez-Moles MA, et al.	2002	Spain	78 OSCC	IHC- LMP-1, PCR- EBV DNA, ISH- EBER- EBVRNA	FFPE	EBV DNA, LMP-1, EBER	Expression of LMP-1 in 12 (85.7%), EBV DNA- 15 (19.2%), EBER- 0 of the EBV-positive OSCC	[57]
Shimakage M, et al.	2002	Japan	37 OSCC	ISH	FFPE	EBV DNA	EBV DNA detected in large number of OSCCs (72%) obtained by nucleotide sequence analysis.	[58]
Ikuta K, et al.	2000	Japan	141 (48 throat washings from healthy adults and 93 salivas from healthy children)	PCR	Saliva	EBV DNA	EBV DNA detected in 90% (43 of 48) in throat washings from healthy adults. (38%) (35 of 93) in saliva from healthy children.	[59]
Tsuhako K, et al.	2000	Japan	102 (60 OSCC from Okinawa and 42 OSCC from Sapporo)	PCR	FFPE	EBV DNA	EBV DNA detected in 76.67% (46 of 60 cases) of OSCC were positive for EBV in Okinawa. 38.1% (16 of 42 cases) of OSCCS were positive for EBV in Sapporo.	[60]
Kobayashi I, et al.	1999	Japan	46 OSCC	EBV DNA by combination of PCR and southern blot hybridization method. LMP-1 by IHC. EBER-1 by ISH	FFPE	EBV DNA, LMP-1, EBER-1	EBV DNA detected in 15.2% (7 out of 46) samples LMP1 was detected in 13% (6 out of 46) samples. EBV-encoded small RNA (EBER)-1 was not demonstrated in any of the sample.	[61]
Gonzalez-Moles M, et al.	1998	Spain	108 OSCC	PCR	FFPE	EBV DNA	EBV DNA detected in 26.3% (5 of 19 cases) well differentiated OSCCs 73.7% (14 of 19 cases) moderately and poorly differentiated OSCCs. (Percentage positivity of EBV DNA increases from well differentiated OSCC to poorly differentiated OSCC)	[62]
D’ Costa J, et al.	1998	India	279 (103 OSCC, 100 OPMD, and 76 NM)	PCR	FFPE	EBV DNA	EBV DNA detected in NM- 4% (3 of 76) OSCC 25% (25 of 103) Oral lesions (OL, OLP, oral submucous fibrosis, melanoplakia, erythroplakia)-13% (13 of 100)	[63]
Cruz I, et al.	1997	Netherland	48 (12 normal mucosa, 9 premalignant lesions, 36 OSCCs)	PCR	FFPE	EBV DNA	EBV DNA detected in OSCC-100% Premalignant lesions-77.8% NM-8.3%	[64]
Van Heerden WE, et al.	1995	South Africa	120 (90 OSCC, 30 NM)	PCR	FFPE	EBV DNA	EBV DNA detected in -OSCC—24% -Tumour tissues (non-specified)-24% (11 out of 45) -Normal mucosa 37% (11 out of 30)	[65]
Van Rensburg EJ, et al.	1995	South Africa	143 (57 OSCC with fragment of normal tissue, 48 OSCC only, 28 NM)	PCR	FFPE	EBV DNA	EBV DNA detected in -OSCC with a fragment of normal epithelium—25% (14 out of 57) -OSCC without normal epithelium- 27% (13 out of 48) -NM—42% (16 out of 38)	[66]
Mao EJ, et al.	1993	England	80 (20 OSCC, 15 NM)	PCR	Exfoliated Cancer cells	EBV DNA	EBV DNA detected in NM—25% (15 out of 60) OSCC—50% (10 out of 20).	[67]

## Data Availability

Not applicable.
